# MetaNet: a scalable and integrated tool for reproducible omics network analysis

**DOI:** 10.1093/bioinformatics/btag321

**Published:** 2026-05-20

**Authors:** Chen Peng, Liuyiqi Jiang, Zinuo Huang, Xin Wei, Xiaoping Zhu, Zhen Liu, Qiong Chen, Xiaotao Shen, Peng Gao, Chao Jiang

**Affiliations:** MOE Key Laboratory of Biosystems Homeostasis & Protection, and Zhejiang Key Laboratory of Molecular Cancer Biology, Life Sciences Institute, Zhejiang University, Hangzhou, Zhejiang 310058, China; State Key Laboratory for Diagnosis and Treatment of Infectious Diseases, National Clinical Research Center for Infectious Diseases, First Affiliated Hospital, Zhejiang University School of Medicine, Hangzhou, Zhejiang 310009, China; MOE Key Laboratory of Biosystems Homeostasis & Protection, and Zhejiang Key Laboratory of Molecular Cancer Biology, Life Sciences Institute, Zhejiang University, Hangzhou, Zhejiang 310058, China; State Key Laboratory for Diagnosis and Treatment of Infectious Diseases, National Clinical Research Center for Infectious Diseases, First Affiliated Hospital, Zhejiang University School of Medicine, Hangzhou, Zhejiang 310009, China; MOE Key Laboratory of Biosystems Homeostasis & Protection, and Zhejiang Key Laboratory of Molecular Cancer Biology, Life Sciences Institute, Zhejiang University, Hangzhou, Zhejiang 310058, China; State Key Laboratory for Diagnosis and Treatment of Infectious Diseases, National Clinical Research Center for Infectious Diseases, First Affiliated Hospital, Zhejiang University School of Medicine, Hangzhou, Zhejiang 310009, China; MOE Key Laboratory of Biosystems Homeostasis & Protection, and Zhejiang Key Laboratory of Molecular Cancer Biology, Life Sciences Institute, Zhejiang University, Hangzhou, Zhejiang 310058, China; State Key Laboratory for Diagnosis and Treatment of Infectious Diseases, National Clinical Research Center for Infectious Diseases, First Affiliated Hospital, Zhejiang University School of Medicine, Hangzhou, Zhejiang 310009, China; MOE Key Laboratory of Biosystems Homeostasis & Protection, and Zhejiang Key Laboratory of Molecular Cancer Biology, Life Sciences Institute, Zhejiang University, Hangzhou, Zhejiang 310058, China; MOE Key Laboratory of Biosystems Homeostasis & Protection, and Zhejiang Key Laboratory of Molecular Cancer Biology, Life Sciences Institute, Zhejiang University, Hangzhou, Zhejiang 310058, China; State Key Laboratory for Diagnosis and Treatment of Infectious Diseases, National Clinical Research Center for Infectious Diseases, First Affiliated Hospital, Zhejiang University School of Medicine, Hangzhou, Zhejiang 310009, China; MOE Key Laboratory of Biosystems Homeostasis & Protection, and Zhejiang Key Laboratory of Molecular Cancer Biology, Life Sciences Institute, Zhejiang University, Hangzhou, Zhejiang 310058, China; State Key Laboratory for Diagnosis and Treatment of Infectious Diseases, National Clinical Research Center for Infectious Diseases, First Affiliated Hospital, Zhejiang University School of Medicine, Hangzhou, Zhejiang 310009, China; Singapore Phenome Center, Lee Kong Chian School of Medicine, Nanyang Technological University, Singapore, 636921, Singapore; School of Chemistry, Chemical Engineering and Biotechnology, Nanyang Technological University, Singapore, 637371, Singapore; Department of Environmental Health and Department of Molecular Metabolism, Harvard T.H. Chan School of Public Health, Boston, MA 02115, United States; MOE Key Laboratory of Biosystems Homeostasis & Protection, and Zhejiang Key Laboratory of Molecular Cancer Biology, Life Sciences Institute, Zhejiang University, Hangzhou, Zhejiang 310058, China; State Key Laboratory for Diagnosis and Treatment of Infectious Diseases, National Clinical Research Center for Infectious Diseases, First Affiliated Hospital, Zhejiang University School of Medicine, Hangzhou, Zhejiang 310009, China

## Abstract

**Motivation:**

Network analysis has become a central strategy for dissecting complex biological and environmental systems, particularly as modern omics technologies generate increasingly large and heterogeneous datasets. However, current tools often lack the scalability, flexibility, and native multi-omics support required for high-dimensional data analysis. We developed MetaNet, a high-performance R package that unifies network construction, visualization, and analysis across diverse omics layers.

**Results:**

MetaNet enables fast and scalable correlation-based network construction for datasets with more than 10 000 features, providing over 40 layout algorithms, rich annotation utilities, and visualization options compatible with both static and interactive platforms. It further offers comprehensive topological and stability metrics for in-depth network characterization. Benchmarking shows that MetaNet delivers up to a 100-fold improvement in computation time and a 50-fold reduction in memory usage compared to existing R packages. We demonstrate its utility through two representative applications: (1) longitudinal microbial co-occurrence networks revealing airborne microbiome dynamics, and (2) an integrative exposome–transcriptome network of over 40 000 features, uncovering distinct regulatory impacts of biological and chemical exposures. By offering a robust, reproducible, and biologically informed framework, MetaNet advances multi-omics network analysis across biological, ecological, and environmental domains.

**Availability:**

MetaNet package is freely available at https://github.com/Asa12138/MetaNet.

## Graphical Abstract

**Graphical Abstract:**
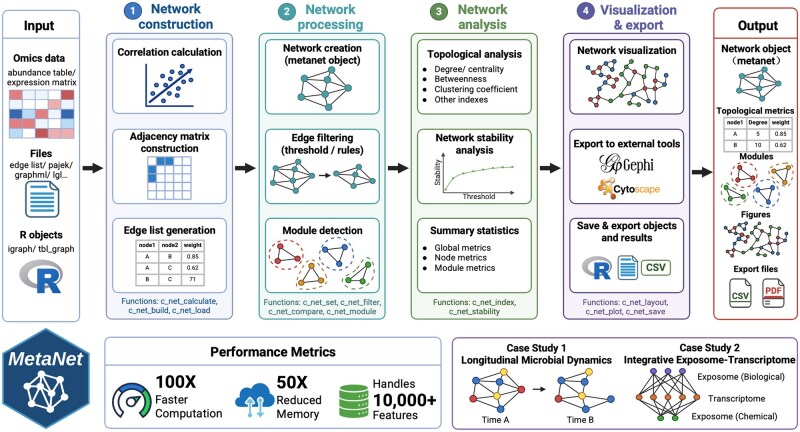

## 1 Introduction

Networks, or graphs, are fundamental tools for modeling relationships in complex biological and environmental systems (Zitnik *et al.* 2024). They provide an abstract yet highly informative representation of interactions among diverse entities—from molecules and cells to microbial communities and environmental factors (Koutrouli *et al.* 2020). Network theory has profoundly influenced numerous subfields of life and environmental sciences, enabling system-level interpretations beyond individual molecular events. Protein–protein interaction (PPI) networks illuminate cellular responses to physiological and toxicological stimuli (Gan *et al.* 2025); co-expression networks capture coordinated gene activity and modular organization of transcriptomes (Liu *et al.* 2023); gene regulatory networks describe hierarchical control in development and disease (Bravo González-Blas *et al.* 2024); metabolic networks map biochemical reactions and energy fluxes (Küken *et al.* 2022); and ecological networks elucidate species interactions and community dynamics (Wu *et al.* 2022). With the explosion of high-throughput omics technologies—such as metagenomics, transcriptomics, proteomics, and metabolomics—network-based approaches have become central to unraveling complex biological, clinical, and environmental dynamics (Wei *et al.* 2022, Chen *et al.* 2024). These networks help reveal modular structures, infer functional associations, and identify key regulators underlying diverse biological and disease processes (Liu *et al.* 2021, Wang *et al.* 2023, Kajihara and Hynson 2024).

A wide range of tools have been developed for network analysis and visualization. Cytoscape offers a user-friendly platform for visualizing molecular interactions, while Gephi provides efficient layout algorithms for large graphs. The igraph, ggraph, and tidygraph packages deliver flexible functions in R and Python; WGCNA is widely used for weighted gene co-expression analysis(Langfelder and Horvath 2008); and tools like ggClusterNet (Wen *et al.* 2022), microeco (Liu *et al.* 2021), CMiNet (Aghdam and Solís-Lemus 2026), and NetCoMi (Peschel *et al.* 2021) extend capabilities for microbiome-specific analyses. Several web-based pipelines [e.g. MENAP (Deng *et al.* 2012) and iNAP (Feng *et al.* 2022)] offer fast, accessible solutions for simple use cases.

However, most existing tools fall short of meeting the requirements of contemporary biological and environmental research. First, few offer native support for multi-omics integration, limiting their ability to uncover cross-layer associations such as links between molecular, microbial, and environmental signals. Second, computational performance is often a bottleneck—especially for correlation-based network construction on high-dimensional datasets, which may require hours or even days (Wen *et al.* 2022). Third, threshold selection for correlation filtering is often subjective and arbitrary, leading to unstable network topology and potentially biased interpretation. Fourth, visualization capabilities are frequently limited, restricting users’ ability to annotate complex networks or to produce publication-ready figures. Finally, many pipelines, particularly web-based tools, lack reproducibility due to unstable computing environments, opaque workflows, or the lack of standardized outputs (Ziemann *et al.* 2023). While such tools remain valuable for general network analysis, they fall short for omics-scale data that require integrative modeling, scalable computation, objective methodology, flexible visualization, and reproducible research.

To address these challenges, we developed MetaNet, a comprehensive and scalable R package tailored for network analysis of omics and multi-omics data. MetaNet fills these gaps through a unified framework that integrates heterogeneous omics layers, enabling the construction of biological, ecological, and exposome-response networks. By leveraging optimized and parallelized algorithms, it achieves fast correlation-based network construction even on datasets with tens of thousands of features. To improve objectivity, MetaNet incorporates random matrix theory (RMT) for data-driven correlation thresholding, enhancing the reliability of network topology (Luo *et al.* 2006). Its extensive visualization module provides over 40 layout algorithms, annotation support, and compatibility with ggplot2, Gephi, and Cytoscape—empowering users to generate customizable, high-quality figures. MetaNet also emphasizes reproducibility, offering curated datasets, step-by-step tutorials, and stable version control with seeded randomness. Finally, the package includes a broad suite of topological and stability metrics for in-depth network interpretation. We demonstrate MetaNet’s capabilities through two representative case studies: a longitudinal airborne microbial co-occurrence network and an integrated exposome–transcriptome network, showcasing its effectiveness for complex, large-scale biological and environmental omics data.

## 2 Methods

### 2.1 Concept design and development of MetaNet

**Figure 1:**
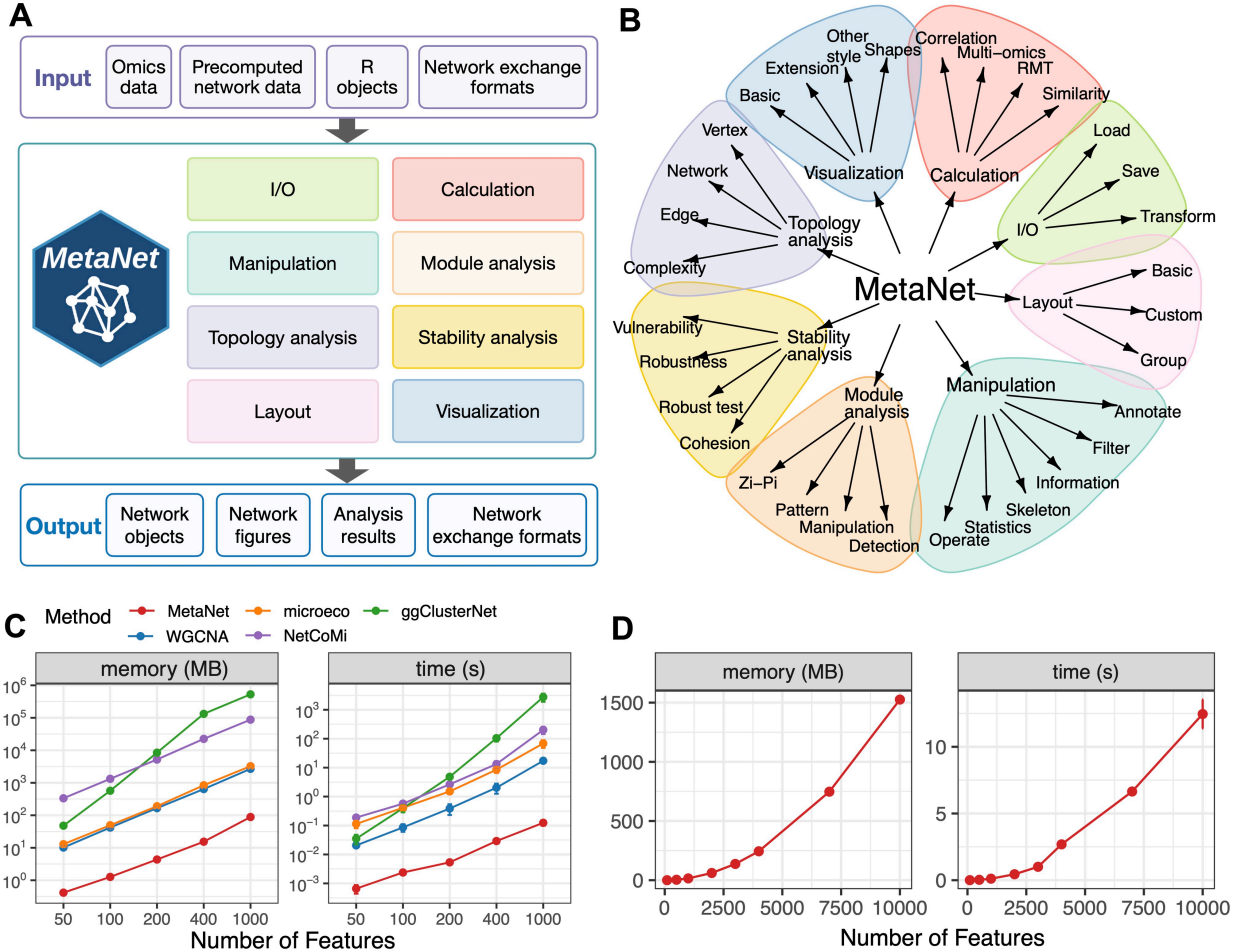
Overview of the MetaNet workflow and its high-efficiency computation. (A) Overview of the MetaNet workflow. The framework accepts multiple input formats, core analytical modules perform network construction, manipulation, module detection, topology analysis, stability assessment, layout generation, and visualization. (B) Functional modules of MetaNet, as visualized using MetaNet. (C) Line plots comparing memory usage and runtime for correlation-based network construction across different R packages. Comparisons were capped at 1000 features because some packages required too many resources and time to process larger networks. Error bars represent standard deviation (SD). (D) Line plots showing MetaNet’s performance on increasingly larger datasets in terms of memory usage and runtime. Error bars represent SD.

MetaNet is an R-based integrative package developed for comprehensive network analysis and visualization across diverse omics datasets, including multi-omics data. It is compatible with Windows, macOS, and Linux systems running R version 4.0 or higher, and its core functionality is built upon the widely used igraph framework. MetaNet adopts a modular architecture encompassing calculation, manipulation, layout, visualization, topology analysis, module analysis, stability analysis, and input/output, thereby supporting an end-to-end workflow from network construction to downstream analysis and visualization (Fig. 1A and B). The central data structure is the “metanet” object, an extension of the igraph class that remains fully compatible with standard igraph operations and can be seamlessly converted into a “tbl_graph” object for integration with the ggraph and tidygraph ecosystems (Table S1). A consistent and intuitive function naming convention, with all core functions prefixed by “c_net_,” facilitates efficient and reproducible analysis.

MetaNet provides extensive data preprocessing capabilities through the “trans” function, offering multiple normalization strategies tailored to different omics types, such as CPM or log-transformation for transcriptomics, aCPM or presence/absence encoding for microbiome data, and log1-transformation for proteomics and metabolomics, complemented by utility functions for feature filtering, cleaning, and merging. Network construction can be achieved directly from raw data, imported from external formats (e.g. GraphML or Pajek), generated from edge lists, or converted from existing igraph objects. Annotation and attribute management are supported through dedicated functions for assigning and retrieving node-, edge-, and network-level metadata, while subnetwork extraction, neighborhood analysis, highlighting, and set-based network comparisons enable flexible network manipulation.

MetaNet further provides access to over 40 layout algorithms, of which 13 are newly developed by MetaNet (e.g. as_polygon, as_polycircle), while the others are enhanced and optimized updates of existing algorithms from widely used R packages such as igraph and ggraph. MetaNet also supports geometric transformations of layouts, including scaling, rotation, mirroring, and pseudo-3D effects, with visualization implemented through a unified plotting interface. Advanced topological characterization is available via computation of 17 commonly used network metrics, and network robustness and structural stability can be evaluated using multiple assessment strategies, particularly suited for ecological and microbial networks. MetaNet is fully open source and publicly available through CRAN, GitHub, and Gitee, is actively maintained in accordance with CRAN policies, and is accompanied by a comprehensive online manual to support both fundamental network analysis and advanced applications.

### 2.2 Comparison with MetaNet and other existing tools

To show the computational efficiency, we compared MetaNet with several widely used R packages for correlation network construction. The “MetaNet::c_net_calculate” function achieves high performance by leveraging vectorized matrix operations with “stats::cor()” and analytically computing *P*-values using a t-distribution formula, avoiding explicit loops for efficiency. Specifically, we benchmarked the following functions: “MetaNet::c_net_calculate” (v0.2.5), “WGCNA::corAndPvalue” (v1.71), “microeco::trans_network$new” (v0.13.1), “ggClusterNet::corMicro” (v2.00), and “NetCoMi::netConstruct” (v1.1.0). Datasets with varying numbers of features (specifically 50, 100, 200, 400, and 1000) were used to evaluate memory usage and computation time for each method. These metrics were measured using the “bench::mark” function (v1.1.2), and each test was repeated 20 times to ensure reliability. Statistical comparisons of memory usage and computation time were performed using the Wilcoxon rank-sum test, revealing significant improvements of MetaNet over other R packages (*P* < 0.001). All benchmark tests were conducted under same conditions: equivalent parameters were used for the core correlation functions of each package, and all tests were performed in a consistent computational environment (macOS with M2 chip, 16 GB RAM, R 4.2.2). The complete, reproducible code for the performance comparison is provided in the “Comparison code” section of the Supplementary Materials.

### 2.3 Case study

We utilized a recently published longitudinal multi-omics dataset that included transcriptomic profiles, biological exposome (microbial exposome), and chemical exposome profiles, collected from individuals in an underwater environment (Huang *et al.* 2024). To study microbial association networks, we first selected microbial taxa with a prevalence threshold of more than 10%. This filtering resulted in a total of 914 microbial species retained for network construction. Species co-occurrence networks were built using Spearman correlation. Only edges with an absolute correlation coefficient (|ρ|) greater than 0.6 and BH-adjusted *P*-values less than 0.05 were retained (Röttjers and Faust 2018). We employed a fast greedy modularity optimization algorithm to evaluate the modularity structure (Clauset *et al.* 2004). Each module represents a set of taxa with stronger intra-group than inter-group connectivity, reflecting potential ecological coherence. To capture temporal variation in microbial association patterns, we extracted sample-specific subnetworks of bacteria based on presence. We calculated a suite of topological indices of subnetworks to characterize the structure of microbial communities over time. These metrics included edge density, proportion of negative edges, average degree, average path length, network diameter, clustering coefficient, eigenvector centrality, betweenness centrality, closeness centrality, degree centrality, and natural connectivity.

For the integrative multi-omics network analysis, correlation networks were constructed between the exposome (biological and chemical) and the transcriptome. For each pair of omics datasets, correlation matrices were computed by calculating Spearman’s rank correlation coefficients and associated *P*-values. To retain only robust associations consistent with the previous study, variable pairs with |ρ| > 0.6 (for chemical–transcriptome pairs) or > 0.5 (for biological–transcriptome pairs) and BH-adjusted *P*-values < 5e–4 were included in the final networks. Finally, for genes significantly associated with either microbial or chemical exposures, we conducted functional enrichment analysis using over-representation analysis (ORA) against the KEGG (Kanehisa *et al*. 2025) and Gene Ontology (The Gene Ontology Consortium *et al.* 2023) (GO) databases by the ReporterScore (v0.2.2) package (Peng *et al.* 2024). This allowed us to identify molecular pathways and biological processes associated with environmental exposures.

## 3 Results

### 3.1 Efficient and scalable network computation enables analysis of larger omics datasets in MetaNet

Network analysis has become a cornerstone of many omics disciplines. Before constructing networks, different omics data types—including microbiome, transcriptome, proteome, and metabolome—require appropriate preprocessing to ensure data quality. MetaNet offers a broad range of normalization strategies (Table S2, see Methods) to support preprocessing across omics types. For example, transcriptomic data can be transformed using methods such as CPM or log-transformation; microbiome data can be normalized using approaches like aCPM (asinh counts per million) or presence/absence (pa) encoding; and mass spectrometry-based proteomics and metabolomics data can be log1-transformed to reduce skewness and stabilize variance. Network construction begins with computing pairwise relationships using statistical strategies. The primary approaches include similarity or correlation-based methods such as “Spearman,” “Pearson,” and “Bray-Curtis.” These generate feature similarity matrices, followed by randomization-based significance testing and multiple-testing correction to retain only meaningful associations. Users can apply common correction methods—including Benjamini–Hochberg FDR, Bonferroni, and Holm—all implemented in “c_net_calculate.”

Pairwise correlation computation is central to most network-based omics tools, but the growing scale of omics datasets imposes substantial computational demands. MetaNet addresses this through optimized vectorized matrix algorithms for calculating correlation coefficients and corresponding *P*-values, greatly reducing memory use and runtime (Fig. 1C, see Methods). Benchmarking showed that MetaNet completed correlation-based analysis on datasets with fewer than 1000 features in under 0.2 seconds and using less than 100 MB of memory (Fig. 1C), outperforming other tools by 100- to 10 000-fold in speed (*P* < 0.001). While other tools may take over an hour on large datasets, MetaNet maintained low resource usage, with memory and runtime scaling approximately quadratically with feature number (Fig. 1D). These efficiencies make MetaNet well-suited for high-throughput network construction.

Correlation-based association networks are widely adopted due to their simplicity and robustness, but threshold selection remains subjective. Many studies rely on manual cutoffs (e.g. |r| > 0.6 and *P* < 0.05) (Röttjers and Faust 2018), introducing inconsistencies and potential biases. To address this, MetaNet incorporates RMT, a statistically grounded method for identifying optimal thresholds. RMT automatically determines a correlation cutoff that minimizes spurious edges based on the data structure, providing a data-driven approach to define the r_threshold parameter for network construction (Fig. S1A and B). While MetaNet primarily supports correlation-based methods, it is compatible with results from alternative inference approaches, including mutual-information methods for non-linear relationships and partial correlations for controlling indirect associations.

### 3.2 Advanced network layout and visualization support in MetaNet

**Figure 2:**
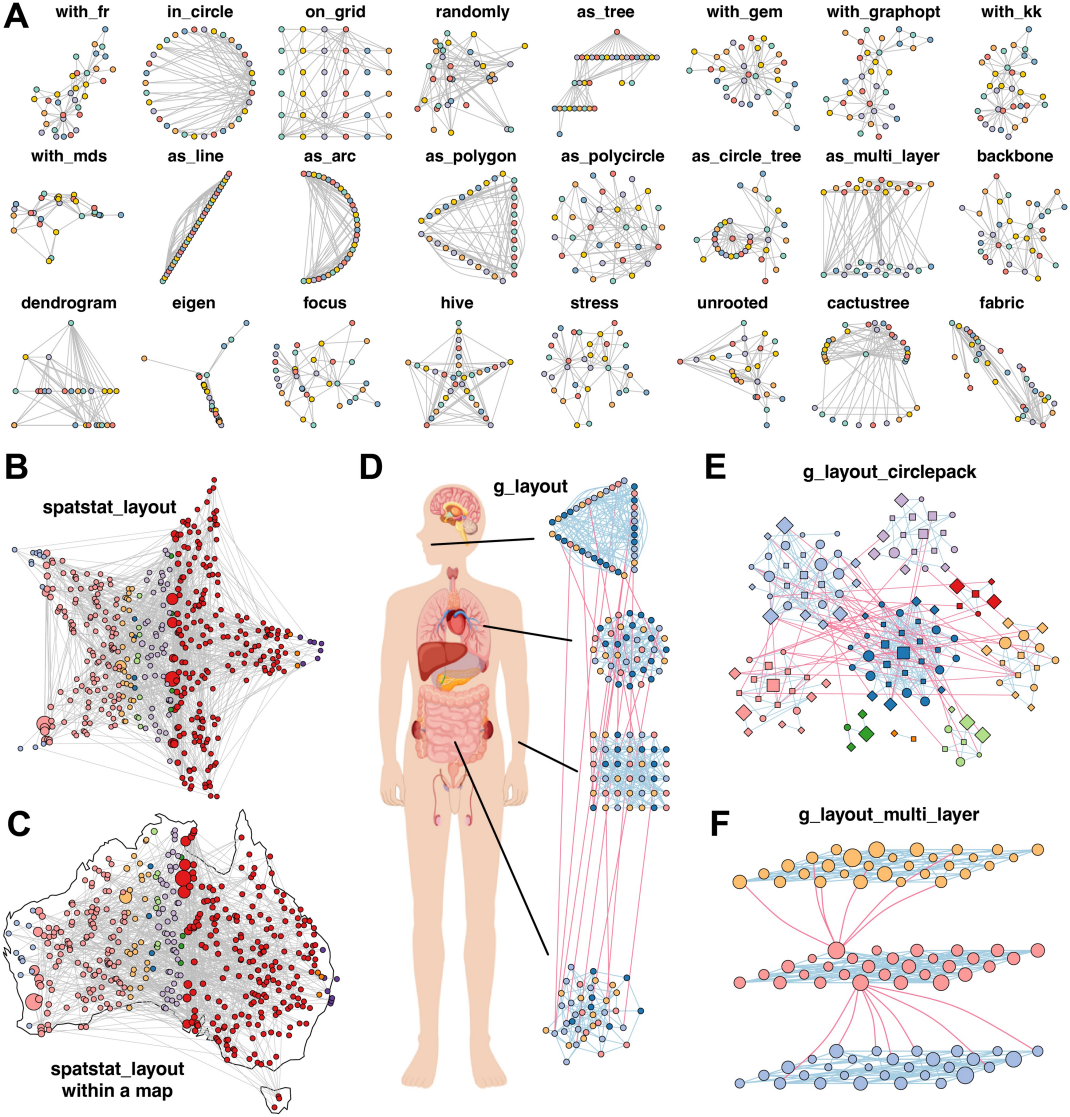
MetaNet enables diverse and powerful network layout strategies. (A) The application of 24 out of more than 40 built-in layout algorithms from “c_net_layout” on the Zachary Karate Club network was provided by the igraph package. (B) Layout generated within a star using “spatstat_layout.” (C) Layout generated within the map of Australia using “spatstat_layout.” (D) Grouped network layout consisting of four subgroups arranged with “with_fr(),” “on_grid(),” “as_polycircle(3),” and “as_polygon(3)” within a human-body schematic. All visualization elements were rendered with MetaNet without manual adjustments. (E) Modular network visualized using “g_layout_circlepack.” (F) A three-layer modular structure visualized using “g_layout_multi_layer.” All networks shown are based on simulated data.

Layout is a critical component of network visualization, as a well-designed layout can significantly enhance the interpretability of network structures (Fruchterman and Reingold 1991). MetaNet stores layout coordinates in a flexible “coors” object, allowing users to control, reuse, and transfer layout settings. The “c_net_layout” function provides access to over 40 layout algorithms (Fig. 2A), including 13 new layouts as well as adaptations from igraph and ggraph packages. In addition to conventional layouts, MetaNet introduces the “spatstat_layout” method, which constrains layout generation within a user-defined polygon or along its edges. This layout function supports uniform or random node distributions inside custom shapes. For example, arranging a network within a star (Fig. 2B) or mapping it to a geographic region like Australia (Fig. 2C). MetaNet also offers interoperability with interactive visualization platforms such as Gephi and Cytoscape, allowing users to import externally generated or manually adjusted layouts.

For networks with grouping variables, MetaNet offers an advanced interface via “g_layout.” Users can define spatial configurations for each group, including positioning, scaling, and internal layout strategies, and combine multiple layout types in one visualization. The resulting “coors” object can be nested or recombined with subsequent calls to create highly customized multi-level layouts. For example, a co-abundance network across multiple human body sites can be arranged with a single “g_layout” call (Fig. 2D). This strategy is also useful for highlighting modular structures. “g_layout_circlepack” visualizes module distribution using compact circular packing (Fig. 2E), while “g_layout_multi_layer” introduces a pseudo-3D representation emphasizing inter-module relationships (Fig. 2F). Additional group layouts are shown in Fig. S2.

MetaNet’s “c_net_plot” function provides extensive parameters for visual customization (Table S3), enabling precise control over nodes, edges, modules, and legends. By default, MetaNet uses base plotting from igraph, but users preferring ggplot2 can convert networks using “as.ggig,” enabling the use of ggplot2 functions such as “labs,” “theme,” and “ggsave” (Fig. S3A). MetaNet also supports exporting visual content to tools like NetworkD3, Gephi, and Cytoscape for extended visualization workflows (Fig. S3B–D).

### 3.3 MetaNet supports flexible network analysis, extended biological network types, and comprehensive topology and stability assessment

MetaNet provides streamlined functional tools for network annotation, manipulation, and comparison, enabling efficient exploratory analysis after network construction. Network, node, and edge attributes can be conveniently accessed and summarized, while annotated network objects remain fully compatible with existing igraph- and tidygraph-based workflows. MetaNet supports flexible network annotation, sub-network extraction, module detection, and cross-network comparison, facilitating focused analysis of specific regions, modules, or conditions within complex omics and multi-omics datasets (Fig. S4, see Supplementary results).

Beyond generic correlation-based networks, MetaNet offers extended support for specialized and database-linked biological network types commonly used in bioinformatics. These include set-based networks, hierarchical tree structures, multivariate node representations, and externally curated biological networks such as protein–protein interactions, regulatory networks, and pathway-based networks. MetaNet also integrates with external functional analysis tools and biological knowledge bases, enabling direct visualization and exploration of pathway-level and regulatory relationships, thereby expanding its applicability across diverse biological contexts (Fig. S5, see Supplementary results).

MetaNet further provides comprehensive tools for network topology and stability analysis, supporting quantitative characterization of both global structure and node-level importance. It enables systematic evaluation of structural properties, modular organization, and topological roles, as well as comparison with randomized networks to assess structural significance. In addition, MetaNet incorporates multiple stability and robustness metrics to model network resilience, vulnerability, and community cohesion, offering insights into the robustness of biological and ecological systems under perturbation (Figs. S6 and S7, see Supplementary results).

### 3.4 Case 1. Longitudinal dynamics of a microbial co-occurrence network

To demonstrate the flexibility of MetaNet in diverse and integrated omics analyses, we applied it to a recently published individual-level longitudinal study involving multi-omics data (Huang *et al.* 2024). In this study, the research team developed wearable passive samplers to perform high-resolution temporal profiling of both chemical and biological exposomes in 19 individuals exposed to a specialized environment. The data included integrated transcriptome and exposome profiles, offering a unique opportunity to examine the effects of environmental perturbations on individual health. Here, we focused on the microbial exposome component, representing the airborne microbiota encountered by each participant over time. Timepoint A represented baseline conditions in a natural environment, while timepoints B to D were recorded in the exposure environment.

**Figure 3:**
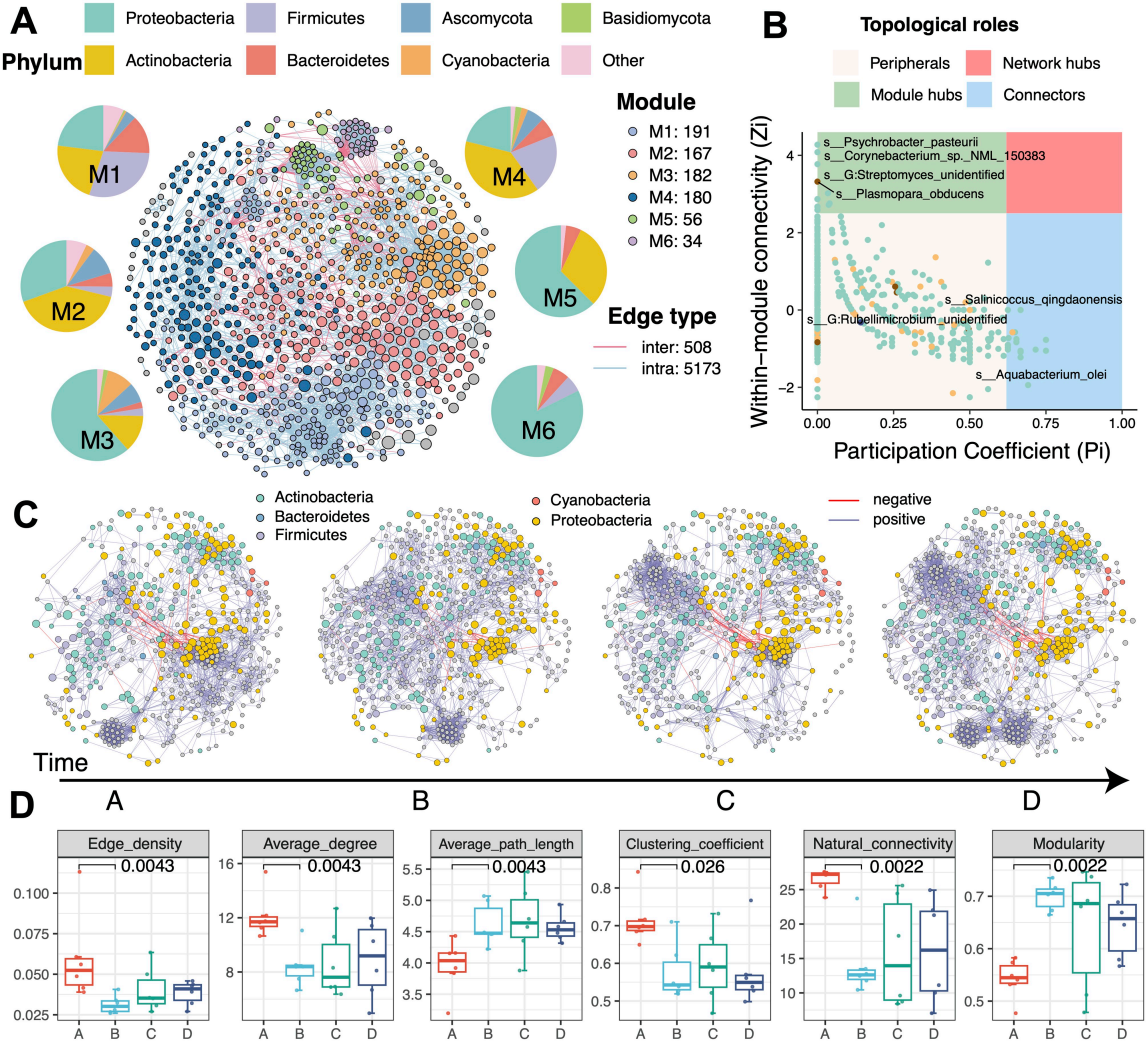
Modularity and temporal dynamics of the microbial co-occurrence network. (A) Species-level microbial co-occurrence network constructed from all microbial exposure samples, showing six modules (M1 to M6). Node color indicates module membership, node size reflects relative abundance, and edge color distinguishes intra- versus inter-module connections. (B) Key microbial taxa identified based on topological role classification using the Zi-Pi framework. (C) Subnetwork dynamics across four exposure stages. Node color represents bacterial phylum, node size reflects relative abundance, and gray nodes denote non-core species with presence or abundance changes over time. (D) Changes in global network topological metrics across different stages. *P*-values for comparisons between timepoints A and B were calculated using the Wilcoxon rank-sum test.

We first constructed a global microbial co-occurrence network (Fig. 3A), which included 871 microbial species spanning four taxonomic kingdoms (Fig. S8A). Using a greedy modularity optimization algorithm (Clauset *et al.* 2004), we identified six distinct modules with varying intra-module species compositions (Fig. 3A). The degree distribution of this network followed a power-law distribution, indicating scale-free properties (Fig. S8B). This suggests that the observed network possesses characteristics of a complex system. Within each module, we analyzed microbial abundance patterns across timepoints. For example, members of Module M3 showed a consistent decline in relative abundance over time. Topological role classification using the Zi-Pi method revealed 13 module hubs and 19 connectors, likely critical for network integrity and inter-module communication (Fig. 3B and Fig. S8C).

We also extracted sub-networks of bacteria for each exposure timepoint (Fig. 3C). A subset of microbial species was found to change in presence or abundance over time (colored in gray). Further topological analysis indicated major shifts from timepoint A to B (Fig. 3D). Compared to the pre-exposure at timepoint A, networks at timepoint B to D exhibited increased modularity and average path length, accompanied by decreases in global efficiency, clustering coefficient, and natural connectivity. These patterns indicate that MetaNet can capture longitudinal changes in inferred microbial association structures under different exposure conditions. These results suggest exposure-associated changes in the airborne microbial association network, which is broadly consistent with previous evidence that the airborne microbiome was destabilized under the specialized exposure conditions (Röttjers and Faust 2018); however, the functional implications of this restructuring require further validation.

### 3.5 Case 2. Multi-omics integrated network maps distinct transcriptomic links to biological and chemical exposome

**Figure 4:**
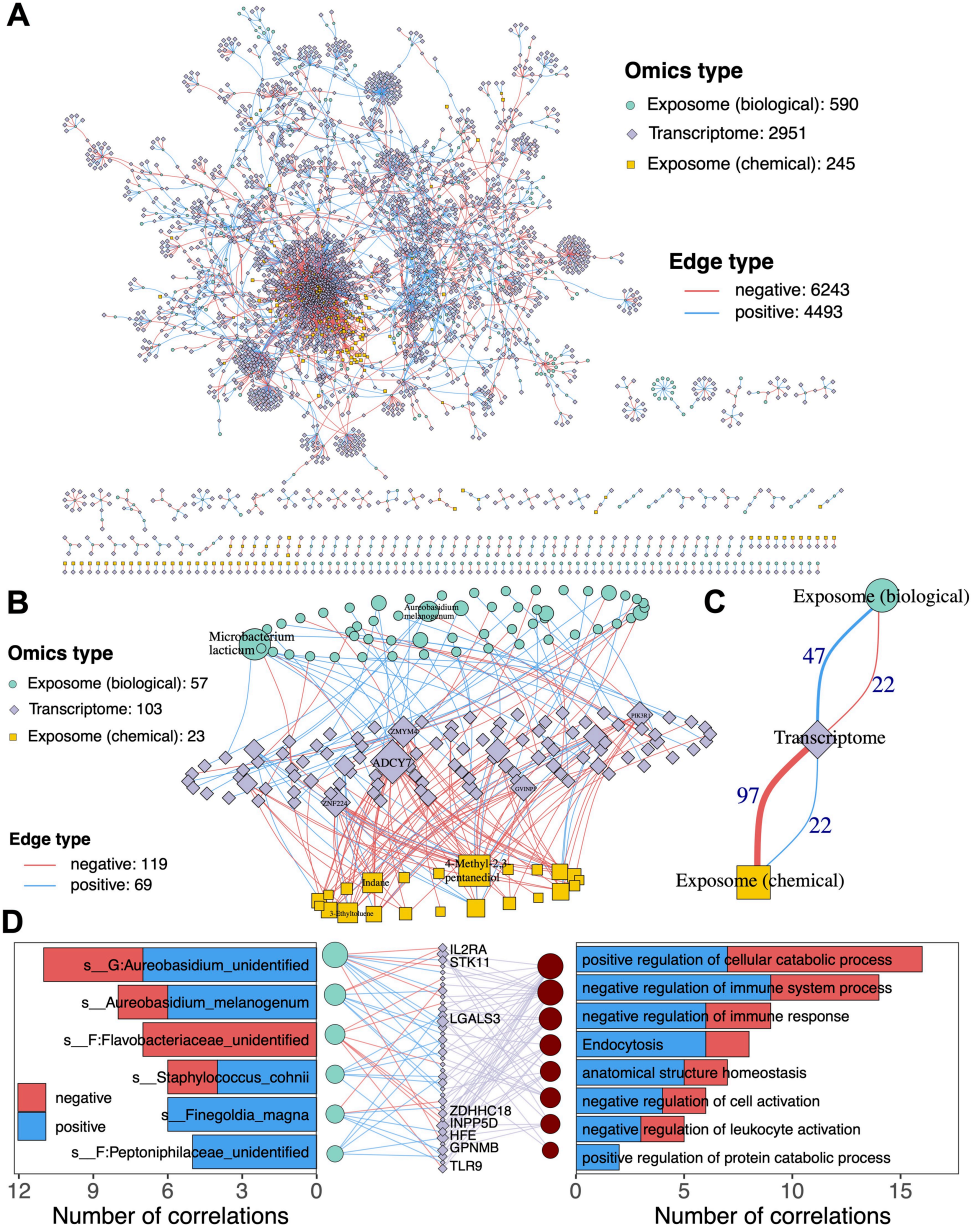
Integrated network analysis of exposome–transcriptome interactions. (A) Spearman correlation-based multi-omics network linking all microbial and chemical exposures to transcriptomic data. (B) Spearman correlation-based multi-omics network showing the most prominent associations. Links with |ρ| > 0.7 (for chemical–transcriptome pairs) or > 0.6 (for biological–transcriptome pairs) are included. Only the top 10 ranked node labels are shown. Node size reflects degree centrality. (C) Skeleton structure of the network in panel B, highlighting the core architecture of microbial and chemical associations with genes. (D) Network representations of significantly correlated genes and enriched pathways for biological exposures. Bar charts on either side indicate the number of positively and negatively correlated connections for each exposure.

We extended our analysis of the longitudinal multi-omics dataset by performing integrated network analysis between the exposome (both biological and chemical) and the host transcriptome. This analysis aimed to characterize the temporal associations between the environmental exposures and gene expression. We used MetaNet to efficiently compute correlation networks between 35 587 transcriptomic genes, 2955 microbial species, and 3729 chemical exposome features (Fig. 4A). Our results revealed that 590 microbial taxa were significantly associated with 1983 genes, with the majority of edges representing positive correlations. In contrast, 245 chemical exposures showed significant associations with 1026 genes, among which negative correlations were more prevalent. Among these, only 58 genes showed significant associations with both microbial taxa and chemical exposures. To visualize the most prominent associations, we presented a multi-omics integrative network based on the strongest correlations (Fig. 4B). The network topology showed a clear distinction: microbial exposures were predominantly positively correlated with gene expression, while chemical exposures showed a bias toward negative associations (Fig. 4C). Among microbial taxa, *Microbacterium lacticum* and *Aureobasidium melanogenum* exhibited the highest number of significant associations with host genes. For chemical compounds, the top contributors included (SR)- or (Rs)-4-methyl-2,3-pentanediol, indole, α,3-dichlorotoluene, and 3-ethylcatechol, with the first compound showing the highest number of gene associations.

We further mapped the genes correlated with either microbial or chemical exposures to the GO and KEGG databases to identify enriched biological functions and pathways (Gao *et al.* 2022). Notably, eight pathways significantly associated with the microbial exposome were identified, most of which were related to the negative regulation of immune responses (Fig. 4D). Among these, several gram-positive anaerobic bacteria showed their positive correlations with genes such as *ADAR*, *GBP1*, and *RHBDF2*, which are key regulators of immune signaling (Grönwall *et al.* 2012). *Streptococcus salivarius*, known for its immunosuppressive properties, showed a significant positively correlation with *HMGB1*, a central mediator in inflammatory signaling, suggesting potential links between microbial exposure and host inflammatory responses (Küchler *et al.* 2013).

In contrast, the chemical exposome was predominantly linked to disease-related pathways (Fig. S9). These included neurodegenerative diseases such as Parkinson’s disease and Alzheimer’s disease, as well as pathways involved in DNA damage response and cellular stress. Some chemicals identified in the dataset, such as benzene, ethylbenzene, and xylene—known to cross the blood–brain barrier—have been implicated in neuropsychiatric disorders, including attention deficits and cognitive decline (Rajendran *et al.* 2022). Polycyclic aromatic hydrocarbons (PAHs), another class of chemicals identified in the dataset, have been associated with elevated risks of DNA damage and carcinogenesis (Poirier 2004). In summary, this case study demonstrates how MetaNet can facilitate integrated multi-omics network analysis by identifying structured association patterns among biological and chemical exposomes and the transcriptome. The divergent correlation structures suggest that microbial and chemical exposures may be associated with different aspects of host transcriptomic variation.

## 4 Discussion

In this study, we present MetaNet, a scalable, flexible, and biologically informed R package for network analysis of omics and multi-omics data. By integrating network construction, visualization, topological analysis, and cross-layer integration into a single reproducible workflow, MetaNet addresses key limitations of existing tools. Its capacity to handle thousands of features, support diverse network types, and generate customizable, high-quality visualizations makes it well-suited for modern multi-omics datasets. The two case studies illustrate MetaNet’s ability to extract meaningful biological and environmental insights, and benchmark comparisons confirm its computational advantages. Its modular design further ensures extensibility and user adaptability.

For microbiome studies, several dedicated tools have been developed. NetCoMi provides a framework tailored for compositional microbiome data with multiple association measures and differential network comparison (Peschel *et al.* 2021), while CMiNet constructs consensus microbial networks by integrating multiple inference algorithms (Aghdam and Solís-Lemus 2026). ggClusterNet and ggNetView emphasize co-occurrence network visualization and modular structure exploration (Wen *et al.* 2022), and microeco offers a broader microbiome analysis workflow where network analysis is not the primary focus (Liu *et al.* 2021). Compared with these tools, MetaNet is designed as a general and scalable framework supporting both microbiome and multi-omics datasets. Although MetaNet currently focuses on correlation-based inference, it provides substantial computational acceleration, flexible layout strategies, and a unified workflow integrating network construction, visualization, topology, and stability analysis. These advantages are illustrated in Case Study 1 through a longitudinal microbial co-occurrence network demonstrating large-scale construction and temporal topology analysis.

Although a variety of network analysis tools are available, each with its own strengths, many face challenges in scalability, multi-omics integration, thresholding selection, or reproducibility—constraints that become particularly limiting for high-dimensional, multi-layer datasets. MetaNet addresses these gaps by offering ultra-fast computation of correlation-based networks, seamless multi-omics integration, comprehensive support for biological and environmental network types, flexible visualization options, and advanced topological and stability metrics. These features make MetaNet particularly well-suited for microbial ecological networks, exposome-driven interaction networks, and studies requiring reproducible visualization. MetaNet also supports specialized biological networks (e.g. KEGG pathways, PPI networks) and continues to expand its application scope. As a fully open-source and extensible framework, MetaNet functions both as a standalone platform and a foundation for multi-omics network pipelines.

Despite these advancements, several limitations remain. MetaNet is currently optimized for correlation-based networks, in which edges represent statistical associations derived from measures such as Spearman correlation. Such networks may capture indirect or spurious associations, particularly in high-dimensional and compositional datasets such as microbiome profiles, where correlations can be affected by data structure and confounding factors. Therefore, network topology and connectivity patterns should be interpreted with caution. Expanding inference strategies—such as partial correlations (Epskamp and Fried 2018) to account for confounding effects, mutual information (Felippe *et al.* 2024) to capture non-linear dependencies, regression-based approaches to model conditional relationships between variables, or compositionality-aware methods such as SparCC—will improve robustness. As omics datasets grow in size and diversity, more efficient algorithms for network construction, layout computation, and topological analysis will be needed. MetaNet also faces challenges common to network analysis (Zitnik *et al.* 2024), including the lack of standardized protocols for similarity metrics, thresholding, and null model selection (Matchado *et al.* 2021). Moreover, commonly used topological metrics (e.g. degree or clustering coefficient) are often intercorrelated, complicating interpretation (Kajihara and Hynson 2024).

MetaNet’s architecture provides fertile ground for future development. Incorporating additional inference methods—such as regression-based, Bayesian, or machine learning approaches—could enhance interpretability. Deeper integration of curated biological knowledge from databases like STRING and KEGG would enable hybrid empirical-prior networks. Supporting sparse matrix formats and network methods tailored to single-cell data would further extend MetaNet’s applicability as single-cell omics expands. Expanding ecological network support, including food webs or host–parasite systems with corresponding metrics, would open new avenues for microbial ecology and environmental omics studies.

## 5 Conclusions

MetaNet provides a scalable, flexible, and reproducible framework for constructing and analyzing networks from high-dimensional omics data. By integrating efficient computation, multi-omics compatibility, and publication-ready visualization, MetaNet addresses critical challenges in modern network analysis. Its open-source and extensible design make it a valuable tool for uncovering regulatory interactions, ecological dynamics, and cross-omics relationships in complex biological and environmental systems. As omics data continue to grow, tools like MetaNet will be essential for enabling interpretable, reproducible, and system-level insights across diverse research domains.

## Supplementary Material

btag321_Supplementary_Data

## Data Availability

Code is available as an open-source R package “MetaNet,” which can be downloaded from GitHub (https://github.com/Asa12138/MetaNet). The main analysis scripts (Rmarkdown format) and source data are available from GitHub (https://github.com/Asa12138/MetaNet_figures). The microbiome data used in the case study are available in the European Nucleotide Archive (ENA) under the accession code PRJEB67959. The processed mass spectrometry datasets, chemical annotation results, species classification information, and transcriptome profile table used in the case study are available from the previously published article’s supplementary materials at: https://pubs.acs.org/doi/suppl/10.1021/acs.est.3c09379/suppl_file/es3c09379_si_002.xlsx.
