# Supplementary material for: MetaNet: a scalable and integrated tool for reproducible omics network analysis
Source: Bioinformatics. 2026 May 20;42(6):btag321. doi: 10.1093/bioinformatics/btag321 (PMC13224960; doi:10.1093/bioinformatics/btag321)
Supplement: btag321_Supplementary_Data [file btag321_supplementary_data.zip › Supplementary_material0423.docx]

# Supplementary results

**Concept Design and Development of MetaNet**

MetaNet is an R-based integrative package designed for comprehensive network analysis across diverse omics data, including multi-omics datasets. MetaNet is compatible with operating systems (Windows, macOS, and Linux) that support R version 4.0 or higher, and its core functionality is built upon the widely used igraph package. Its architecture comprises several core functional modules: Calculation, Manipulation, Layout, Visualization, Topology analysis, Module analysis, Stability analysis, and I/O (Figure 1A), supporting the end-to-end analytical process from network construction to visualization. Figure 1B illustrates the main workflow and essential components within MetaNet. The central data structure in MetaNet is the "metanet" object, which extends the widely used "igraph" class. The "metanet" object is fully compatible with all basic igraph operations and can be converted to a "tbl_graph" object for integration with the ggraph and tidygraph packages. MetaNet provides a streamlined set of functions tailored to the "metanet" object, facilitating easy construction, annotation, manipulation, visualization, and analysis of biological networks. The workflow is organized through a consistent set of logically structured functions, all beginning with the prefix "c_net_", making them easy to remember and apply (Table S1).

**Data preprocessing**

MetaNet provides a broad range of normalization strategies through the "trans" function to accommodate the preprocessing requirements of diverse omics data types (Table S2). For example, transcriptomic data can be transformed using methods such as CPM or log-transformation; microbiome data can be normalized using approaches like aCPM (asinh counts per million) or presence/absence (pa) encoding; and mass spectrometry-based proteomics and metabolomics data can be log1-transformed to reduce skewness and stabilize variance. MetaNet also includes utility functions such as "guolv" and "hebing" for users to screen, clean, and combine raw feature tables before network construction.

**Network object construction**

Networks can be constructed through several approaches. One method is to calculate and construct networks directly from raw omics data using the "c_net_calculate" and "c_net_build" functions. Alternatively, users may import external network files in formats such as graphml or pajek using the "c_net_load" function. Networks can also be built from existing edge list tables using "c_net_from_edgelist". Finally, "c_net_update" allows for upgrading a conventional igraph object into a fully functional "metanet" object.

**Annotation**

Once a network object is constructed, various operations can be performed on this object. Annotation and attribute assignment are facilitated through "c_net_set" and "c_net_annotate", enabling advanced downstream data analysis and visualization. And the "get_*" family of functions retrieves tables of network, node, and edge attributes.

**Manipulation**

To extract subnetworks or specific components, functions such as "c_net_filter", "c_net_neighbors", and "c_net_highlight" can be used. For comparative analysis across networks, "c_net_union", "c_net_intersect", and "c_net_difference" allow for set operations. Community detection or module extraction is achieved using "c_net_module", which enables users to perform module-based analysis.

**Layout**

For any network, whether original or customized, users can obtain flexible and visually informative layouts using "c_net_layout" and "g_layout", which provide access to over 40 layout algorithms. The "transform_coors" function supports various geometric transformations of layouts, including scaling, aspect ratio adjustments, rotation, mirroring, and pseudo-3D. Visualization is accomplished through the "c_net_plot" function, which offers a variety of parameter settings to help users effectively display network structure and attribute information (Table S3).

**Topological analysis**

MetaNet also includes advanced functionality for network topology characterization through the "c_net_index" function, which computes 17 widely used topological metrics. Network robustness and structural stability can be evaluated using the "c_net_stability" function, which incorporates multiple stability assessment strategies, particularly relevant for applications such as microbial ecological networks.

**Accessibility and deployment**

MetaNet is completely open source and publicly available on CRAN ([https://CRAN.R-project.org/package=MetaNet](https://cran.r-project.org/package=MetaNet)), GitHub (<https://github.com/Asa12138/MetaNet>), and Gitee (<https://gitee.com/Asa12138/MetaNet>). It is actively maintained following CRAN policies. A comprehensive online manual is also provided to assist users in learning the basics of network analysis and the detailed usage of MetaNet, available at <https://bookdown.org/Asa12138/metanet_book/>.

## Comparison code

library(bench)
feature_numbers <- c(50,100,200,400,1000)
n_repeats=20
results <- data.frame()
for (rep in 1:n_repeats) {
 for (n in feature_numbers) {
 set.seed(123)
 tmp <- matrix(rnorm(n * 50), ncol = n)%>%abs()
 colnames(tmp) <- paste0("feature_", 1:n)
 mt <- microeco::microtable$new(otu_table = t(tmp))
 ps <- phyloseq::phyloseq(phyloseq::otu_table(as.matrix(tmp), taxa_are_rows = FALSE))
 cat("\n=== Evaluating with", n, "features ===\n")
 bm <- bench::mark(
 MetaNet = MetaNet::c_net_calculate(tmp, method = "spearman"),
 WGCNA = WGCNA::corAndPvalue(tmp, method = "spearman"),
 microeco = {
 microeco::trans_network$new(mt, cor_method = "spearman",
 filter_thres = 0, use_WGCNA_pearson_spearman = FALSE)
 },
 ggClusterNet = {
 ggClusterNet::corMicro(ps, method = "spearman", r.threshold = 0, p.threshold = 1)
 },
 NetCoMi = NetCoMi::netConstruct(tmp, measure = "spearman", thresh = 0, alpha = 1),

 iterations = 1,
 check = FALSE
 )
 temp_df <- data.frame(
 n_features = rep(n,5),
 iteration = rep,
 method = c("MetaNet", "WGCNA", "microeco", "ggClusterNet", "NetCoMi"),
 time = as.numeric(unlist(bm$time)),
 mem_alloc = as.numeric(bm$mem_alloc) / 1024^2
 )
 results <- rbind(results, temp_df)
 }
}

## Streamlined functional tools for network annotation, manipulation, and comparison in MetaNet

Once a network is constructed, exploratory analysis and flexible manipulation are essential for extracting biological insights. MetaNet provides a suite of functions to support these tasks. The “get_*” family retrieves tables of network, node, and edge attributes for inspection and statistical summarization. The resulting “metanet” object is fully compatible with igraph operations and can be converted to a “tbl_graph” object for integration with ggraph and tidygraph.

In omics and multi-omics studies, networks are often annotated with external data such as abundance profiles, taxonomy, or clinical metadata. The “c_net_set” function attaches multiple annotation tables to a network object and automatically configures visualization properties (Figure S4B), including color schemes, line types, node shapes, and legends.

After annotation and customization, researchers may focus on specific network regions—especially in multi-omics integration. The “c_net_filter” function extracts sub-networks using flexible filters (Figure S4C), while “c_net_highlight” visually emphasizes selected nodes or edges (Figure S4D).

Modules or communities—densely connected subgraphs—often represent biologically meaningful groups. MetaNet supports module detection through “c_net_module”, which includes multiple community detection algorithms (Figure S4E). Resulting modules can be visualized with chord or Sankey diagrams to show proportions and inter-module connections (Figure S4F). For group-level analysis, the “c_net_skeleton” function summarizes edge origins and targets across conditions, enhancing interpretability in multi-condition or longitudinal datasets (Figure S4G).

Comparative analysis across multiple networks is also critical. Researchers may identify differential edges between groups or track stable subnetworks across transitions. MetaNet enables such comparisons by computing intersections, unions, and differences between networks (Figure S4H), offering a flexible framework for comparative and evolutionary network analysis.

## Extended support for specialized and database-linked biological networks

MetaNet provides native support for a variety of specialized network types frequently used in bioinformatics workflows, enabling researchers to visualize and explore biological relationships beyond conventional correlation or interaction networks.

MetaNet allows the construction of Venn-style networks to illustrate set relationships across sample groups. These provide a more informative alternative to traditional Venn diagrams by displaying explicit connections and network structure (Figure S5A). Tree-structured data, such as taxonomies or gene ontology hierarchies, can be visualized using the built-in "as_circle_tree" layout, offering a clear and compact representation of hierarchical relationships (Figure S5B). MetaNet further supports pie-node visualization, where each node encodes multivariate annotations, such as group-specific abundances. This approach allows compositional data to be embedded directly in the network structure (Figure S5C).

Beyond generic network types, MetaNet is compatible with biological networks from external databases. For example, protein–protein interaction (PPI) networks obtained from the STRING database can be imported and visualized with customized layout and annotations (Figure S5D). Similarly, miRNA–target gene regulatory networks from miRTarBase, which are experimentally validated, can be represented to explore post-transcriptional regulatory mechanisms (Figure S5E).

MetaNet also integrates with the ReporterScore, an R package we previously developed for functional enrichment analysis. Using the results of pathway enrichment, users can directly visualize relationships between KEGG orthologs (KOs) and their associated pathways (Figure S5F). Furthermore, MetaNet supports direct rendering of any KEGG pathway map through a specified pathway ID, enabling fully annotated and modifiable visualizations (Figure S5G).

Together, these extended features highlight MetaNet’s versatility in accommodating diverse biological network types, integrating with external knowledge bases, and enhancing the interpretability of complex multi-omics analyses.

## Comprehensive network topology and stability analysis in MetaNet

Network topology refers to the structural patterns formed by connections between nodes and edges, reflecting both global architecture and local importance in biological systems^1^. In omics research, topological analysis is crucial for understanding molecular interactions and functional organization. MetaNet offers a broad suite of metrics for characterizing networks at global and local levels. Global metrics describe the overall structure—including density, average degree, clustering coefficient, path length, natural connectivity, and others (Table S4)—quantifying properties such as redundancy, robustness, and signal propagation. For example, average path length represents the typical number of steps needed to traverse a network, indicating signaling efficiency in metabolic or gene regulatory systems^2^. Local metrics evaluate the importance or centrality of individual nodes or edges (Table S5), helping identify key regulators or bottlenecks.

To assess structural significance, MetaNet can generate random networks using the Erdős–Rényi model^3^ with identical numbers of nodes and edges as the observed network (Figure S6A). This allows for comparison with real omics data-derived networks, which often exhibit scale-free, small-world, modular, and hierarchical features^4^. The "fit_power" function tests for scale-freeness by fitting a power-law to the degree distribution (Figure S6B), while "smallworldness" computes the small-world index σ. Modular structure is a hallmark of biological networks, representing clusters of closely connected nodes often corresponding to functional features (Figure S6C). MetaNet implements multiple community detection algorithms via the "c_net_module" function, allowing users to examine expression or abundance patterns within modules (Figure S6D). Using the Zi-Pi method^5^, MetaNet further classifies nodes into four topological roles: peripherals, connectors, module hubs, and network hubs (Figure S6E and S6F).

Beyond topology, network stability is essential in modeling robustness in molecular systems, ecosystems, and metabolic regulation^6^. MetaNet incorporates several algorithms to assess structural and ecological stability, leveraging "parallel::detectCores" for enhanced efficiency. For structural robustness, MetaNet calculates natural connectivity as nodes are progressively removed^7^ (Figure S7A). The rate of decline in connectivity reflects the network’s resilience to perturbation^8^. Robustness is assessed by simulating node removals and tracking survival based on the abundance-weighted mean interaction strength^9^ (Figure S7B). Vulnerability reflects a node’s contribution to global efficiency, indicating its critical role in network communication^9^ (Figure S7C). Cohesion indices, both positive and negative, measure cooperation and competition within microbial communities^10^ (Figure S7D and S7E).

# Supplementary figures


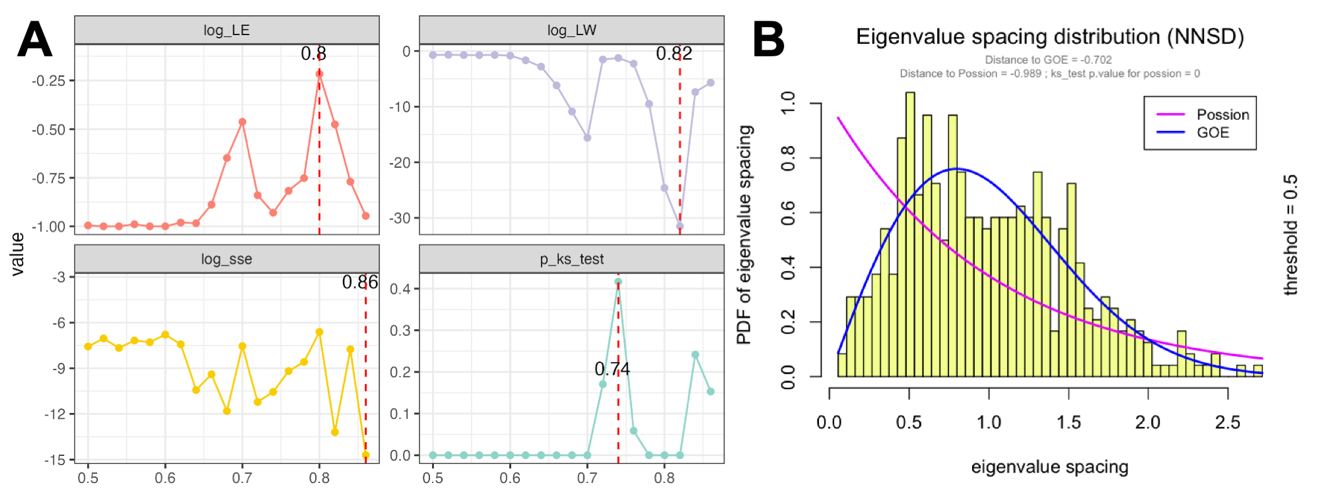


**Figure S1. Evaluation of RMT-based network threshold optimization.**

(A) Line plots showing changes in key random matrix theory (RMT)-based statistics under different correlation thresholds. A meaningful threshold is indicated by higher values of log_LE and p_ks_test, and lower values of log_LW and log_SEE. These metrics help identify the optimal "r_threshold" for robust network construction.

(B) Probability density function (PDF) of eigenvalue spacing distribution when the correlation threshold is set to 0.5.


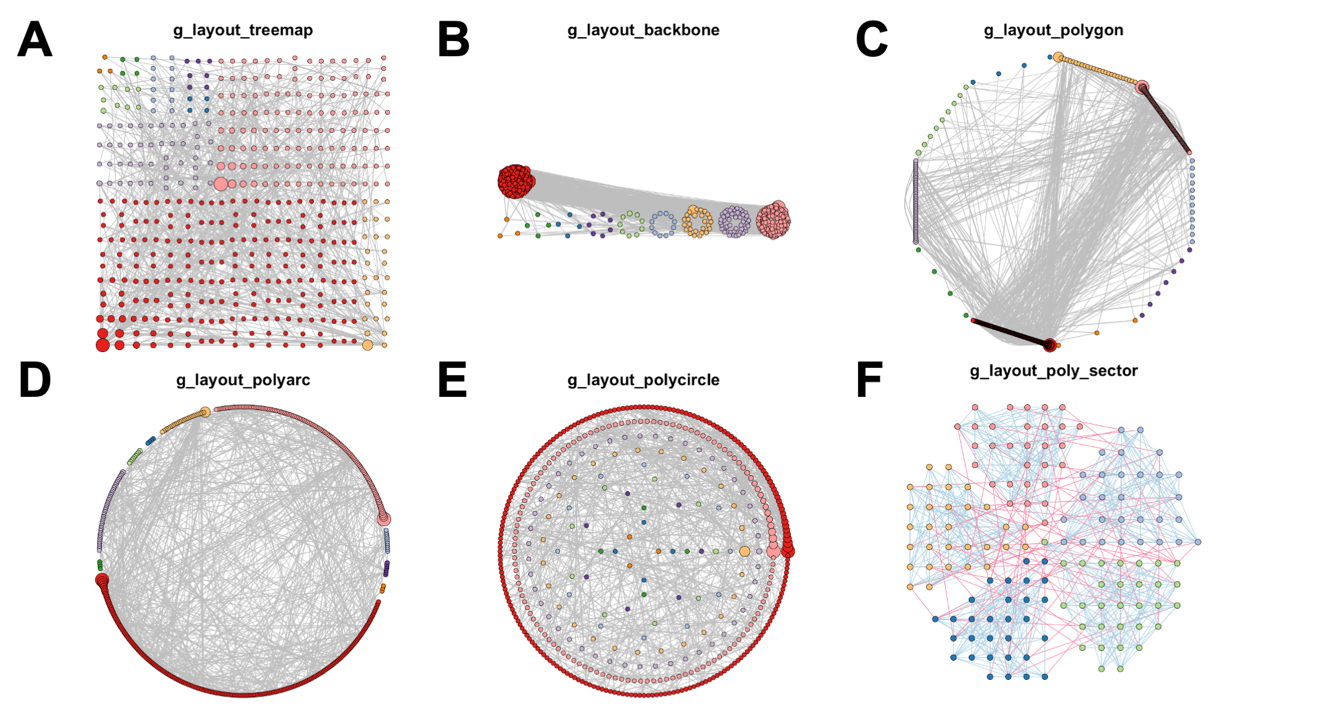


**Figure S2. Preset layout options for group-based network visualization.** (A) g_layout_treemap, (B) g_layout_backbone, (C) g_layout_polygon, (D) g_layout_polyarc (E) g_layout_polycircle, and (F) g_layout_poly_sector.


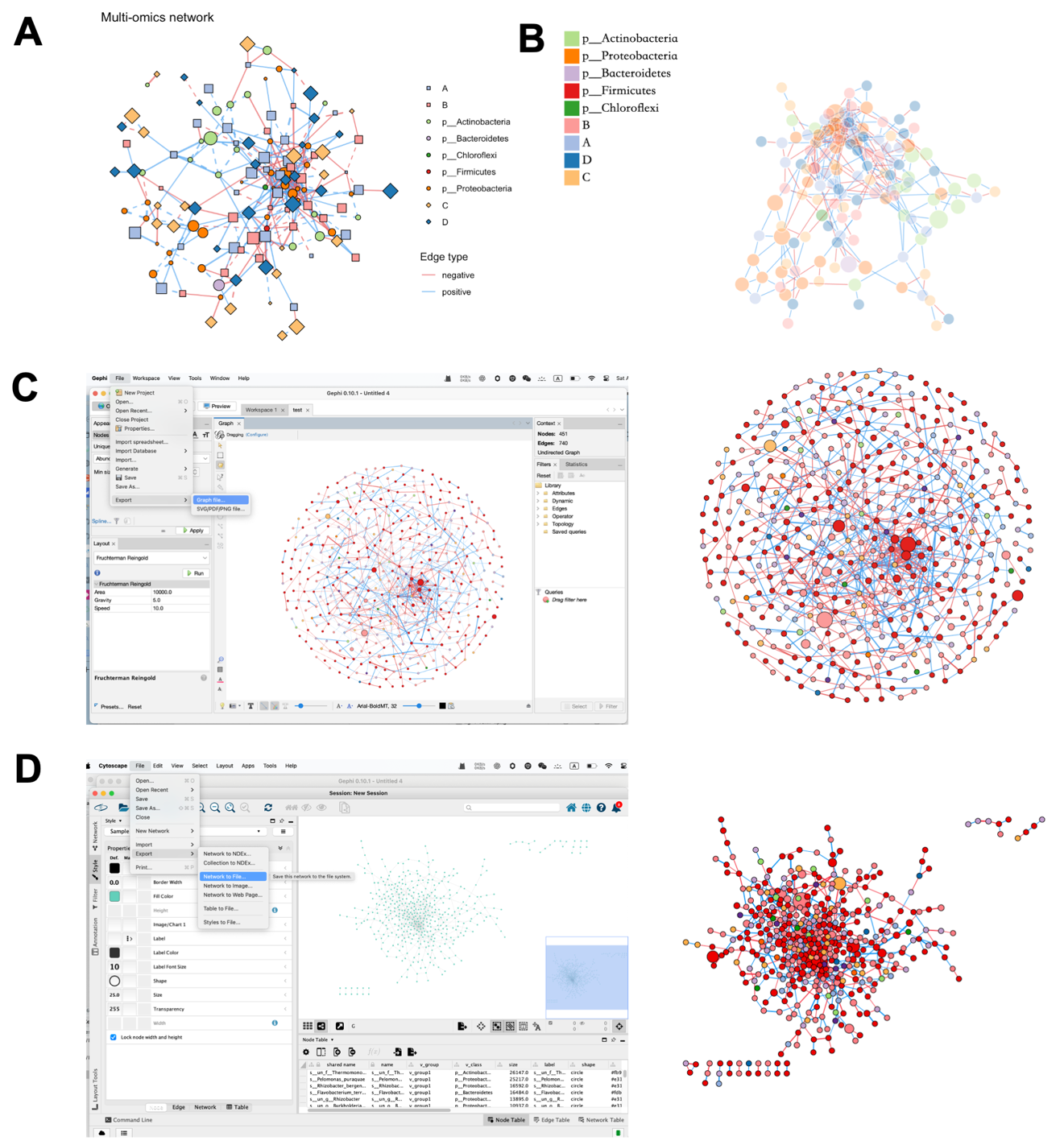


**Figure S3. MetaNet compatibility with external visualization tools.**

Extension of MetaNet to multiple external visualization and analysis platforms, including: (A) ggplot2-based rendering via "as.ggig", (B) D3.js-based interactive networks via " netD3plot", (C) exported static layout rendering in Gephi, and (D) import and annotation within Cytoscape.


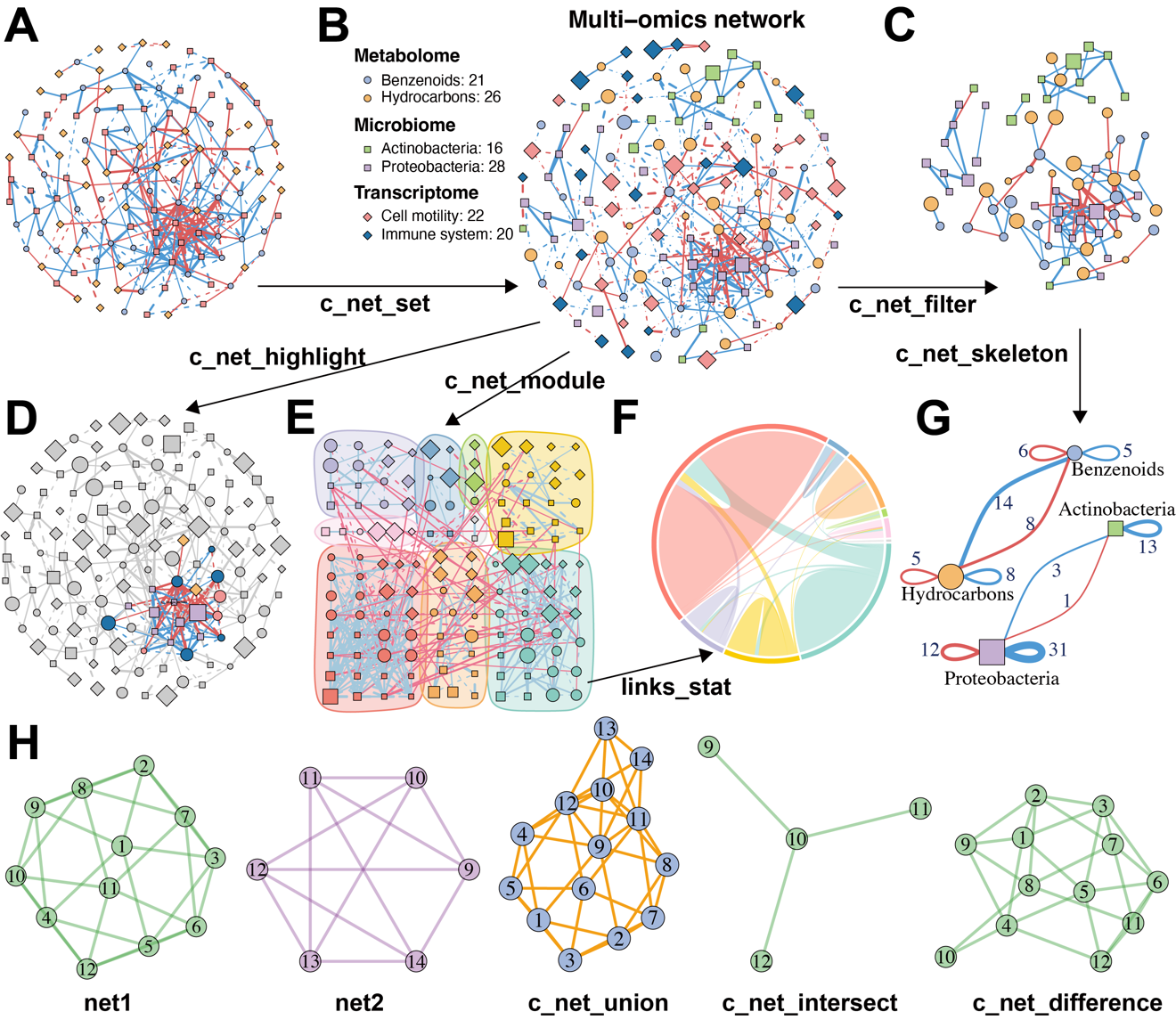


**Figure S4. MetaNet supports flexible and intuitive network manipulation.**

(A) Initial multi-omics network constructed without annotations.

(B) Annotated multi-omics network using the "c_net_set" function. Node shape indicates the types of omics data, color represents the subtypes of omics data, size denotes average abundance, edge color indicates positive or negative correlation, edge type (solid or dashed) distinguishes intra- and inter-omics connections, and edge width reflects the absolute value of the correlation coefficient.

(C) Subnetwork filtered from intra-omics interactions between the Microbiome and Metabolome layers using "c_net_filter".

(D) Highlighted nodes centered on "Dongia_mobilis" and its neighbors using "c_net_highlight".

(E) Community detection and modular visualization using "c_net_module".

(F) Chord diagram displaying the proportion of edges between modules.

(G) Skeleton network across omics subtypes at a grouped level using "c_net_skeleton".

(H) Operations among networks: "c_net_union" merges net1 and net2, "c_net_intersect" extracts shared nodes and edges, and "c_net_difference" isolates net1-specific nodes and edges. All networks shown are based on simulated data and are for illustrative purposes only.


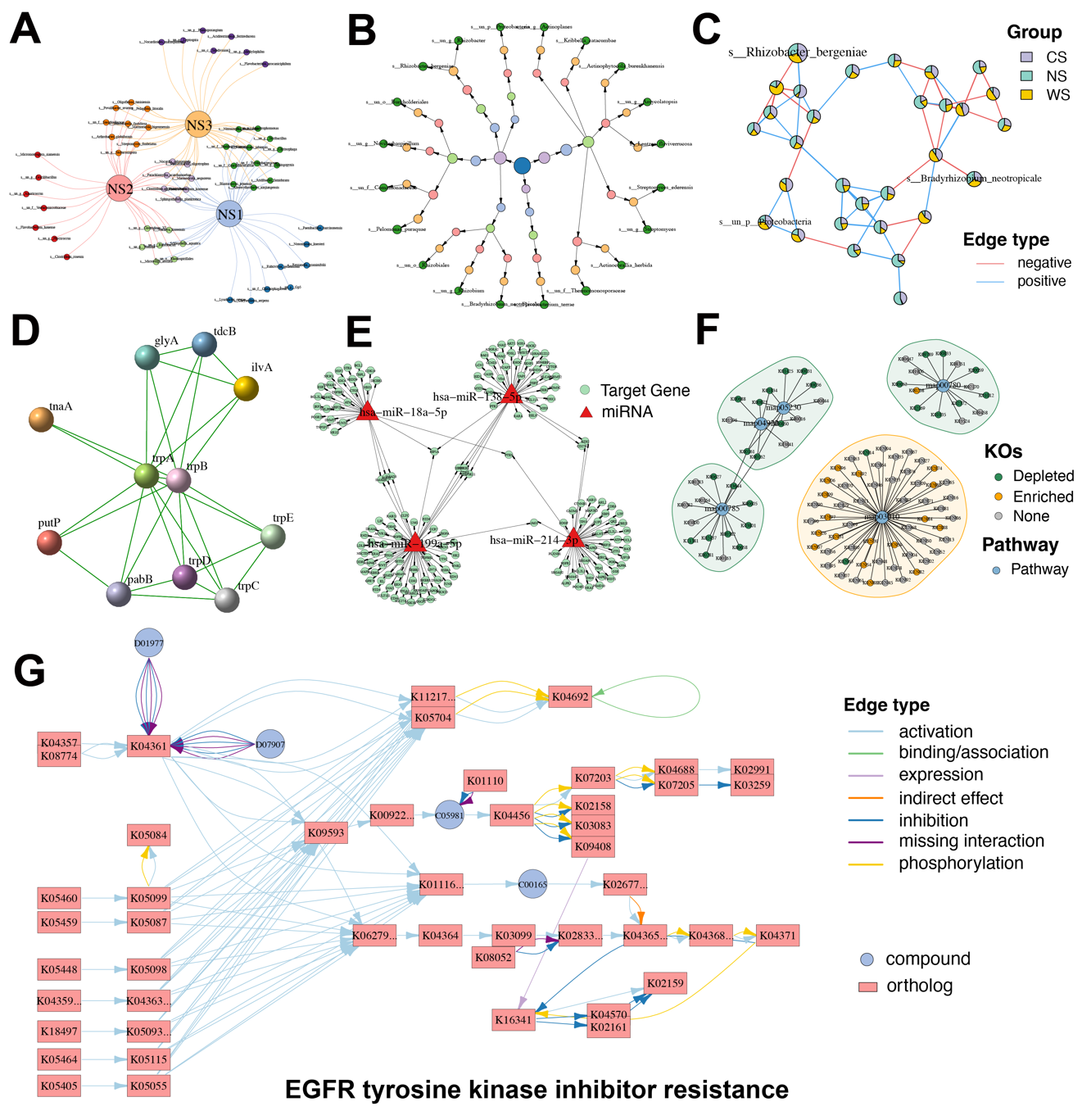


**Figure S5. Diverse specialized network visualizations by MetaNet.**

(A) Venn-style network: Large nodes represent groups, while smaller nodes denote individual elements within each group, enabling visualization of shared and unique components.

(B) Hierarchical tree network: Nodes are organized based on classification hierarchy. Node color corresponds to the taxonomic or categorical level.

(C) Pie-node network: Each node is displayed as a pie chart, where slice colors indicate relative abundance across different groups.

(D) Protein–protein interaction (PPI) network: Extracted from the STRING database, showing experimentally validated and predicted molecular interactions among proteins.

(E) miRNA–gene regulatory network: Sourced from the miRTarBase database, illustrating experimentally supported regulatory relationships between miRNAs and their target genes.

(F) KEGG KO–pathway association network: The network shows KEGG orthologs (KOs) involved in selected biological pathways. Small nodes represent KOs, and large nodes represent pathways. KO nodes are colored by their expression trend. Shaded regions surrounding pathways indicate whether the pathway is globally up-regulated (orange) or down-regulated (green).

(G) KEGG pathway-specific network: Network representation of the “EGFR tyrosine kinase inhibitor resistance” pathway. Rectangular nodes denote KEGG orthologs, circular nodes indicate compounds, and edge colors reflect interaction types.


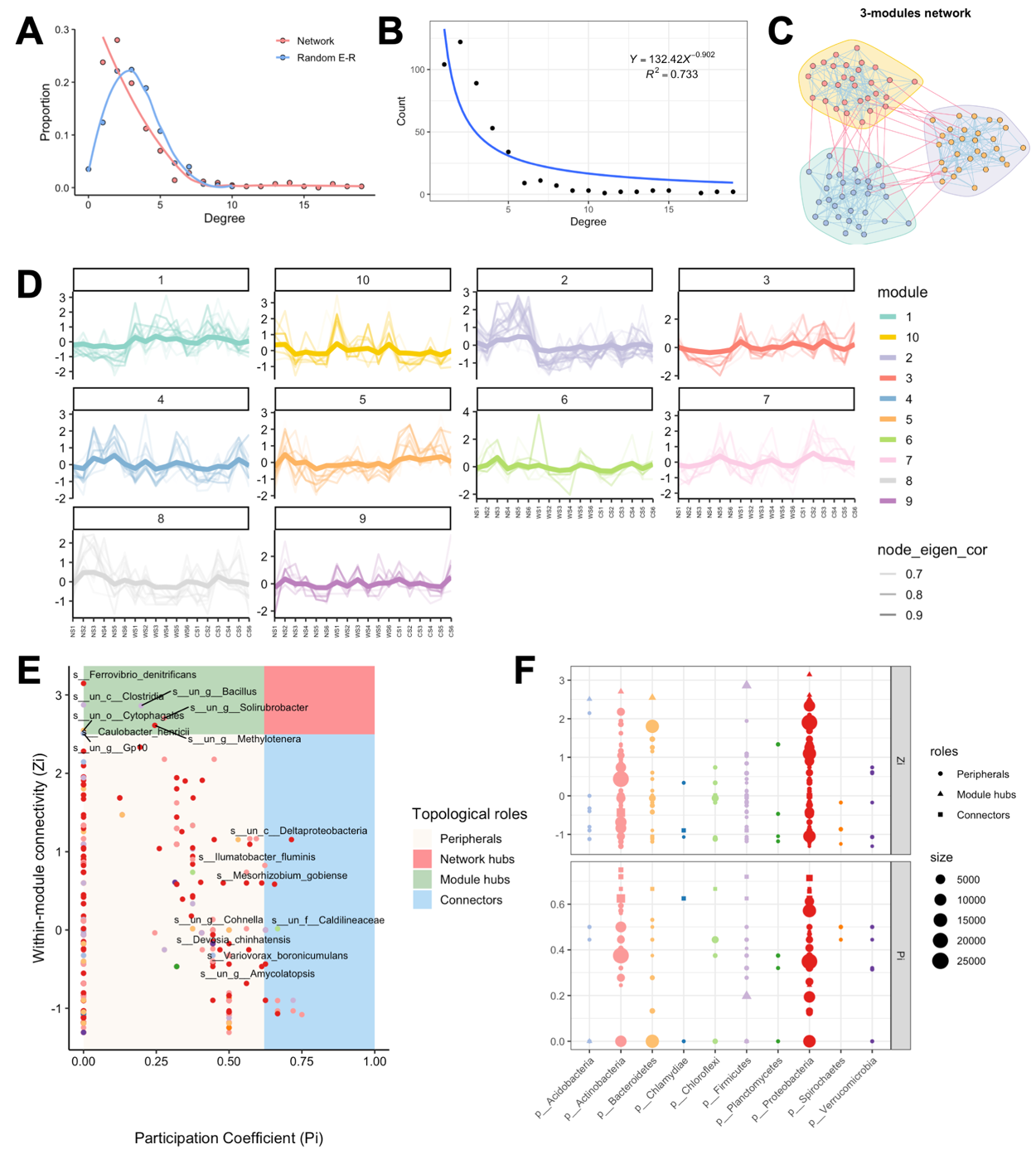


**Figure S6. Structural properties and modular analysis of microbial networks.**

(A) Degree distribution comparison between the constructed microbial network and a random network with the same number of nodes and edges.

(B) Power-law fitting of degree distribution in the constructed network.

(C) Example of a module-level subnetwork extracted from the global network.

(D) Line plots of intra-module species abundance across samples.

(E) Classification of node topological roles using participation coefficient (P) and within-module connectivity (Zi).

(F) Distribution of Zi and P values across different microbial phyla.


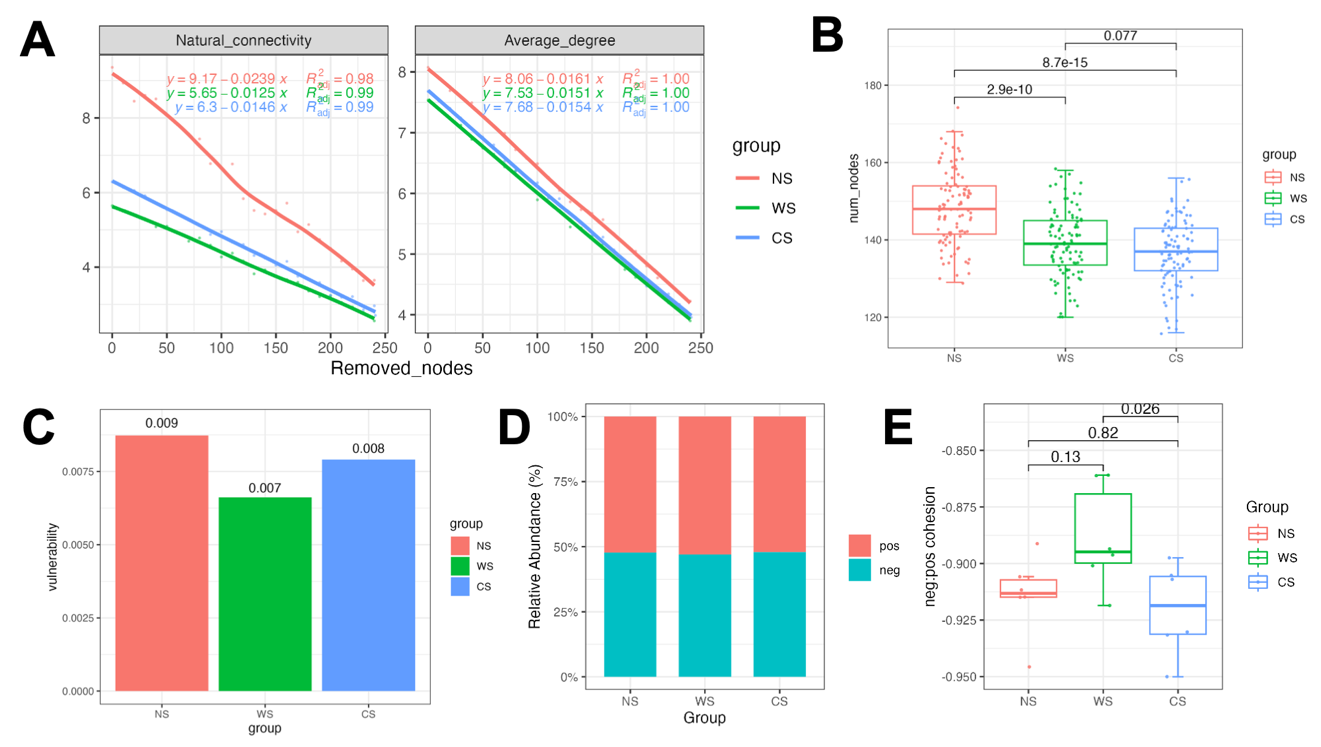


**Figure S7. Network robustness and cohesion metrics.**

(A) Changes in natural connectivity and average degree during node removal simulation in three subnetworks.

(B) Box plots comparing robustness scores of the three subnetworks.

(C) Calculated vulnerability indices for each subnetwork.

(D) Bar plots showing the relative proportions of positive and negative cohesion in the three subnetworks.

(E) Box plots comparing the negative-to-positive cohesion ratios (neg: pos) among the subnetworks.


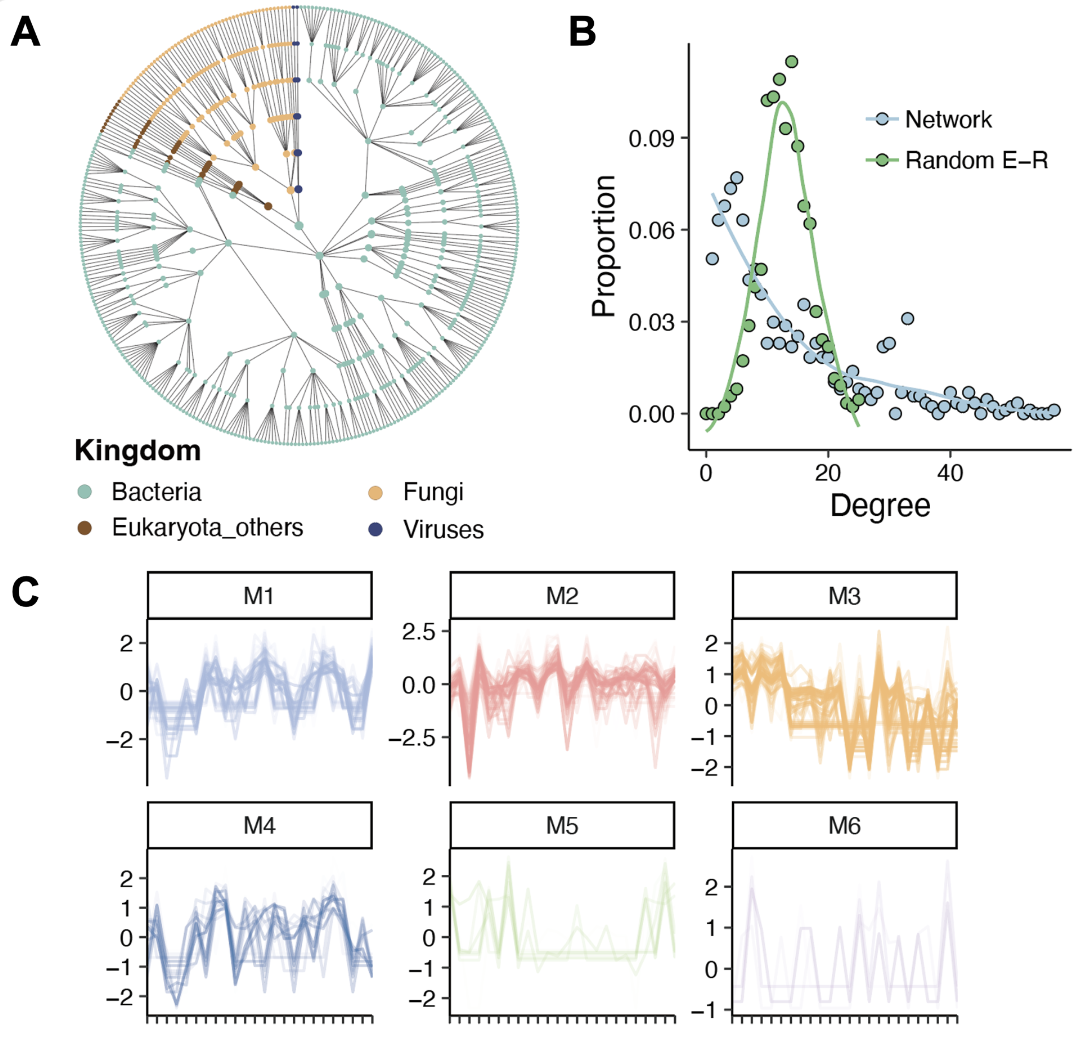


**Figure S8. Modularity and temporal dynamics of the microbial co-occurrence network.**

(A) Phylogenetic relationship network of all species in panel A, arranged using the "as_circle_tree" layout.

(B) Comparison of degree distribution between the empirical network in panel A and a randomized network with the same number of nodes and edges.

(C) Temporal abundance profiles of species within each module. The y-axis represents the scaled abundance of species, while the x-axis represents individual samples sorted by time point.


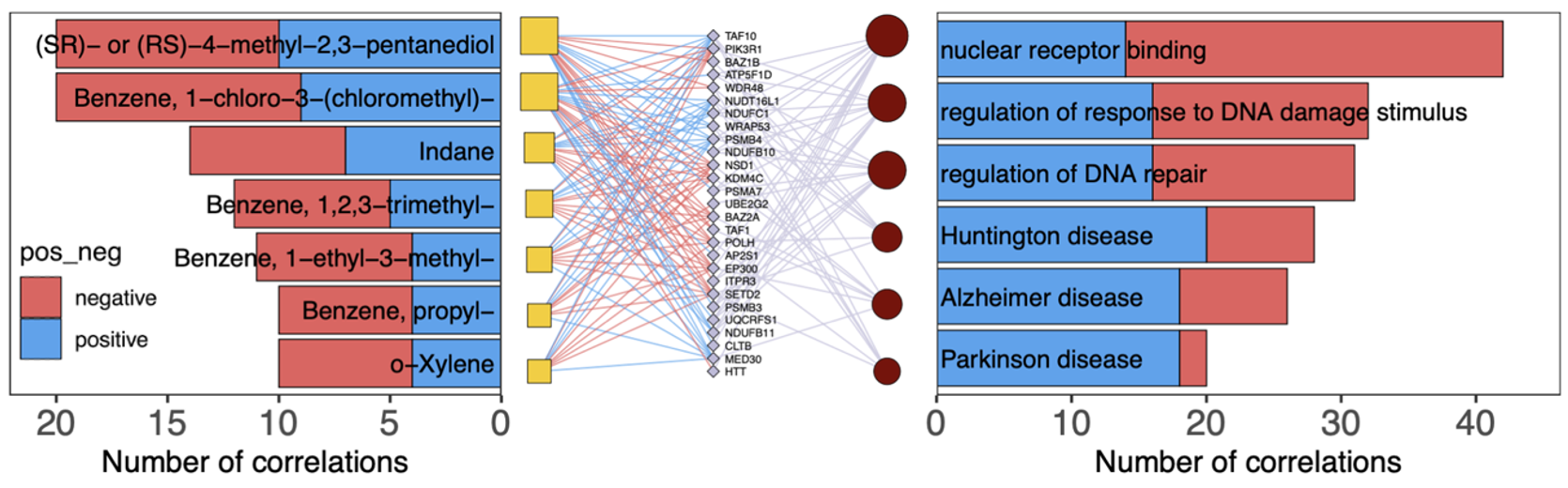


**Figure S9. Correlation networks between the exposome and host transcriptome.** Network representations of significantly correlated genes and enriched pathways for chemical exposures. Bar charts on either side indicate the number of positively and negatively correlated connections for each exposure.

# References

1. Antoniazzi, R., Dáttilo, W. & Rico-Gray, V. A useful guide of main indices and software used for ecological networks studies. in *Ecological Networks in the Tropics: an Integrative Overview of Species Interactions from Some of the Most Species-rich Habitats on Earth* (eds Dáttilo, W. & Rico-Gray, V.) 185–196 (Springer International Publishing, Cham, 2018). doi:10.1007/978-3-319-68228-0_13.

2. Barabási, A.-L. & Oltvai, Z. N. Network biology: understanding the cell’s functional organization. *Nat. Rev. Genet.* **5**, 101–113 (2004).

3. Seshadhri, C., Kolda, T. G. & Pinar, A. Community structure and scale-free collections of Erdo ̋s-Re ́nyi graphs. *Phys. Rev. E* **85**, 56109 (2012).

4. Jiang, W., Ye, W., Tan, X. & Bao, Y.-J. Network-based multi-omics integrative analysis methods in drug discovery: a systematic review. *Biodata Min.* **18**, 27 (2025).

5. Guimerà, R. & Nunes Amaral, L. A. Functional cartography of complex metabolic networks. *Nature* **433**, 895–900 (2005).

6. Kajihara, K. T. & Hynson, N. A. Networks as tools for defining emergent properties of microbiomes and their stability. *Microbiome* **12**, 184 (2024).

7. WU Jun, M. B. Natural connectivity of complex networks. *Chin. Phys. Lett.* **27**, 78902–78902 (2010).

8. Shi, Y. *et al.* Interannual climate variability and altered precipitation influence the soil microbial community structure in a tibetan plateau grassland. *Sci. Total Environ.* **714**, 136794 (2020).

9. Yuan, M. M. *et al.* Climate warming enhances microbial network complexity and stability. *Nat. Clim. Change* **11**, 343–348 (2021).

10. Herren, C. M. & McMahon, K. Cohesion: a method for quantifying the connectivity of microbial communities. https://doi.org/10.1038/ismej.2017.91 (2017) doi:10.1038/ismej.2017.91.
